# What does the fox say? Monitoring antimicrobial resistance in the environment using wild red foxes as an indicator

**DOI:** 10.1371/journal.pone.0198019

**Published:** 2018-05-25

**Authors:** Solveig Sølverød Mo, Anne Margrete Urdahl, Knut Madslien, Marianne Sunde, Live L. Nesse, Jannice Schau Slettemeås, Madelaine Norström

**Affiliations:** Norwegian Veterinary Institute, Sentrum, Oslo, Norway; Natural Environment Research Council, UNITED KINGDOM

## Abstract

The objective of this study was to estimate and compare the occurrence of AMR in wild red foxes in relation to human population densities. Samples from wild red foxes (n = 528) included in the Norwegian monitoring programme on antimicrobial resistance in bacteria from food, feed and animals were included. All samples were divided into three different groups based on population density in the municipality where the foxes were hunted. Of the 528 samples included, 108 (20.5%), 328 (62.1%) and 92 (17.4%) originated from areas with low, medium and high population density, respectively. A single faecal swab was collected from each fox. All samples were plated out on a selective medium for *Enterobacteriaceae* for culturing followed by inclusion and susceptibility testing of one randomly selected *Escherichia coli* to assess the overall occurrence of AMR in the Gram-negative bacterial population. Furthermore, the samples were subjected to selective screening for detection of *E*. *coli* displaying resistance towards extended-spectrum cephalosporins and fluoroquinolones. In addition, a subset of samples (n = 387) were subjected to selective culturing to detect *E*. *coli* resistant to carbapenems and colistin, and enterococci resistant to vancomycin. Of these, 98 (25.3%), 200 (51.7%) and 89 (23.0%) originated from areas with low, medium and high population density, respectively. Overall, the occurrence of AMR in indicator *E*. *coli* from wild red foxes originating from areas with different human population densities in Norway was low to moderate (8.8%). The total occurrence of AMR was significantly higher; χ^2^ (1,N = 336) = 6.53, *p* = 0.01 in areas with high population density compared to areas with medium population density. Similarly, the occurrence of fluoroquinolone resistant *E*. *coli* isolated using selective detection methods was low in areas with low population density and more common in areas with medium or high population density. In conclusion, we found indications that occurrence of AMR in wild red foxes in Norway is associated with human population density. Foxes living in urban areas are more likely to be exposed to AMR bacteria and resistance drivers from food waste, garbage, sewage, waste water and consumption of contaminated prey compared to foxes living in remote areas. The homerange of red fox has been shown to be limited thereby the red fox constitutes a good sentinel for monitoring antimicrobial resistance in the environment. Continuous monitoring on the occurrence of AMR in different wild species, ecological niches and geographical areas can facilitate an increased understanding of the environmental burden of AMR in the environment. Such information is needed to further assess the impact for humans, and enables implementation of possible control measures for AMR in humans, animals and the environment in a true “One Health” approach.

## Introduction

Antimicrobial resistance (AMR) is considered one of the main public health challenges in modern times [1]. The increased emergence of AMR around the world is a result of the selection pressure exerted on the bacterial population by the use of antimicrobial agents [2,3]. Much focus has been given to the occurrence of AMR in humans and different domesticated animal species. However, AMR should be considered a “One Health” problem [4,5] including environmental aspects, as the continuous exchange of bacteria between different environmental niches is likely to contribute to its dissemination [6,7].

The red fox (*Vulpes vulpes*) is common and widespread throughout Norway and its habitat ranges from non-inhabited remote areas to large cities. A recent study of Scandinavian red foxes revealed that their home range is rather limited compare to the total area of Norway and varied between 0.95 km^2^ to 44 km^2^ [8]. Furthermore, the difference in home range size is explained by studies showing that fragmented agricultural landscapes and vicinity to human settlements allow for high prey densities, leading to smaller home ranges of red foxes in urban areas. This enables comparison of differences in the occurrences of AMR among foxes living in areas with different human population densities, possibly reflecting differences in the occurrence of resistance drivers and environmental load of resistant bacteria. Resistance drivers include antimicrobials, but also other substances such as biocides and heavy metals. Genes encoding resistance towards these substances can often be located on the same genetic elements, and therefore exposure to such substances can co-select for several resistance mechanisms, including AMR [9]. It is likely that foxes living in urban areas will come in direct or indirect contact with human infrastructure; such as food waste, garbage, sewage and waste water. Furthermore, red foxes are top predators, and may acquire AMR bacteria through consumption of prey [10]. Thus, the red fox may represent a good sentinel for monitoring AMR occurrence in the environment which could improve our understanding of the dynamics and drivers for resistance in the environment. It is a potential risk of transmission of AMR from the environment to humans and it is therefore of importance to gain knowledge of the environmental burden of AMR to enable targeted measures for risk reduction.

Compared to other European countries the occurrence of AMR in Norway is low, both in the human and veterinary sectors[11].This is documented in the yearly report “Usage of Antimcrobial Agents and Occurrence of Antimicrobial Resistance in Norway (NORM/NORM-VET)”. This report includes data from both the human and veterinary AMR surveillance systems (The Norwegian surveillance programme for antimicrobial resistance in human pathogens (NORM) and the Norwegian monitoring programme on antimicrobial resistance in bacteria from food, feed and animals (NORM-VET). However, knowledge on the environmental reservoirs of AMR in Norway is limited. Thus, we aimed to estimate the occurrence of AMR in wild red foxes in relation to human population densities. We hypothesized that the population density would affect the exposure of wild red foxes to antimicrobials and other potential resistance drivers in addition to AMR bacteria.

## Materials and methods

### Study design

Samples from wild red foxes (n = 528) included in NORM-VET 2016 [12] were available for the present study. A single faecal swab was collected from each fox. The samples were originally collected from red foxes during the hunting season in 2016 under the auspices of the Norwegian monitoring programme for *Echinococcus multilocularis* [13]. The major proportion of the samples was frozen upon arrival at the Norwegian Veterinary Institute. However, in the early phase of the study, some samples were not frozen ahead of analyses.

The samples were allocated to three different groups based on human population density in the municipality where the foxes were hunted. The groups were defined as low, medium and high population density areas; low for municipalities with < 5 inhabitants per km^2^, medium for municipalities with 5–200 inhabitants per km^2^, and high for municipalities with >200 inhabitants per km^2^, thereby also including the largest cities, based on population density data of 2015 derived from Statistics Norway (number of inhabitants per km^2^) (www.ssb.no, accessed 19.09.2016). In order to allow discriminates between municipalities with no or very limited livestock production and municipalities with a higher livestock production density, several additional data sources were used. These included topgraphical data from the Norwegian Mapping Authority (www.kartverket.no), data on livestock population derived from the registry of production subsidies (as of 31.07.2015) and the location of each livestock production unit in Norway from the agricultural registry.

**Fig 1:**
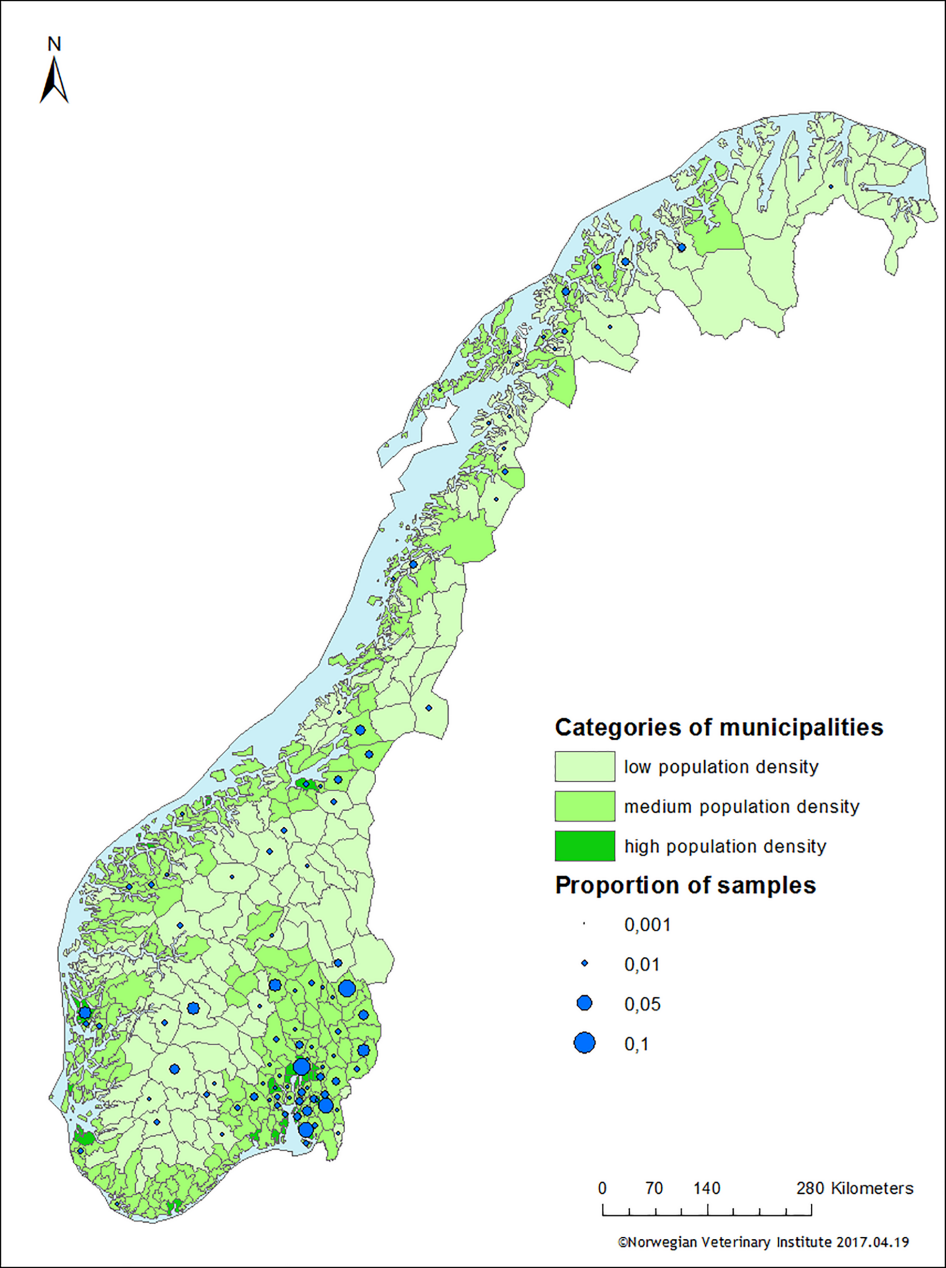
Distribution of sampled wild red foxes per municipality. Each municipality was categorised according to the human population density (in green) and the proportion of samples within each municipality is displayed as blue circles.

The grouping of municipalities and distribution of samples from wild red foxes hunted in each municipality is illustrated in Fig 1. Of the 528 samples in the study, 108 (20.5%), 328 (62.1%) and 92 (17.4%) originated from areas with low, medium and high population density, respectively. A subset of the samples (n = 387) were additionally subjected to supplementary selective screening for detection of carbapenem and colistin resistance. Of these, 98 (25.3%), 200 (51.7%) and 89 (23.0%) originated from areas with low, medium and high population density, respectively.

### Bacterial isolation

The methods described below for isolation of *E*.*coli* and screening of specific resistances have been performed with the recommended methods used for the routine monitoring of resistance in food and animals as reported yearly to EFSA [11] and as performed in NORM-VET.

#### Indicator *E*. *coli*

Faecal swabs were directly plated on MacConkey agar (Difco, Sparks, MD, USA) and incubated at 41°C±0.5°C for 24–48 hours. One colony with typical *E*. *coli* morphology was randomly selected, sub-cultured on blood agar (Oxoid, Basingstoke, UK) and confirmed as *E*. *coli* by a positive indole test.

#### *E*. *coli* resistant to extended-spectrum cephalosporins (ESC), colistin, carbapenems and fluoroquinolones

After direct plating, the faecal swabs were inoculated in 5 mL buffered peptone water (BPW-ISO, Oxoid) and incubated at 37°C±1°C for 20±2 hours. Ten μL of the overnight enrichment was plated on MacConkey agar with 1 mg/L cefotaxime (Duchefa, Haarlem, the Netherlands) and MacConkey agar with 2 mg/L ceftazidime (Sigma-Aldrich) for detection of ESC-resistant *E*. *coli* and on MacConkey agar with 0.06 mg/L ciprofloxacin (Sigma-Aldrich) for detection of fluoroquinolone resistant *E*. *coli*. From a subset of the samples (n = 387), ten μL of the overnight enrichment was also plated on chromID^TM^ CARBA and chromID^TM^ OXA-48 (bioMérieux, Marcy l’Etoile, France) for detection of CPE and on SuperPolymyxin agar [14] for detection of colistin-resistant *E*. *coli*, respectively. MacConkey agar plates and SuperPolymyxin agar plates were incubated at 41°C±0.5°C for 24–48 hours, while chromID^TM^ agar plates were incubated at 37°C±1°C for 24–48 hours. Presumptive *E*. *coli* isolates originating from the selective agar plates were sub-cultured on blood agar and respective selective agar plate before they were confirmed as *E*. *coli* using matrix assisted laser desorption/ionization time of flight mass spectrometry (MALDI-TOF MS, Bruker Daltonics).

#### Vancomycin resistant enterococci

From the 387 subset of samples, faecal swabs were directly plated on Slanetz and Bartley agar (Oxoid) with 4 mg/L vancomycin (Sigma-Aldrich, St.Louis, MO, USA) and incubated at 41°C±0.5°C for 48 hours. Colonies with typical morphology were cultured on blood agar and confirmed as *Enterococcus faecium* or *Enterococcus faecalis* by MALDI-TOF MS.

### Susceptibility testing

All isolates were subjected to antimicrobial susceptibility testing following the protocol used for routine monitoring [11]. Minimum inhibitory concentrations (MICs) were determined by broth microdilution using commercial plates (Sensititre®, TREK diagnostics LTD, Thermo Scientific). Isolates were classified as susceptible or resistant based on epidemiological cut-off values (ECOFFs) recommended by the European Committee on Antimicrobial Susceptibility Testing (EUCAST, www.eucast.org). *E*. *coli* displaying ESC resistance were additionally susceptibility tested using a panel of beta-lactam antimicrobials in order to determine the beta-lactam resistance phenotype (EUVSEC2, Sensititre®). Susceptible *E*. *coli* ATCC 25922 were included as quality control.

### Detection of resistance genes

*E*. *coli* displaying resistance towards ESC or colistin were subjected to PCR for detection of specific resistance genes. ESC-resistant *E*. *coli* displaying an AmpC phenotype (i.e cefoxitin resistance and no synergy with clavulanic acid) were subjected to a RT-PCR for detection of *bla*_CMY_ with previously published primers and probe [15]. If *bla*_CMY_ was not present, the isolates were subjected to a multiplex PCR for detection of plasmid-mediated AmpC genes (pAmpC) *bla*_MOX_, *bla*_CIT_, *bla*_DHA_, *bla*_ACC_, *bla*_EBC_ and *bla*_FOX_ [16] and PCR for detection of mutations in the promoter/attenuator region of the chromosomal *ampC* gene [17]. ESC-resistant *E*. *coli* with an extended spectrum betalactamase (ESBL) phenotype were subjected to PCR for detection of *bla*_TEM_, *bla*_SHV_ and *bla*_CTX-M_ genes [18,19]. Colistin-resistant isolates were subjected to a multiplex PCR for detection of the plasmid-mediated colistin resistance genes *mcr-1* and *mcr-2* [20,21]. PCR amplicons were sequenced to determine the gene responsible for the resistance genotype. Positive and negative controls were included in each PCR run.

### Conjugation experiments

In order to determine if ESC resistance genes were located on transferrable plasmids, conjugation experiments were performed with a subset of ESC resistance isolates (all isolates with an ESBL/pAmpC pheno- and genotype as described previously) [22]. Presumptive transconjugants were subjected to PCR as described above to confirm transfer of the plasmid carrying the relevant resistance gene.

### Data processing

Management and analysis of data were performed in SAS-PC system® version 9.4 for Windows (SAS Institute Inc., Cary, NC, USA). Calculation of 95% confidence intervals for the obtained resistance frequencies was done using the binomial test in R version 3.3.1 for Windows [23]. The χ^2^ or Fisher’s exact test, the latter for small sample sizes, were used to determine whether there was a significant difference in occurrence of AMR between areas with low, medium and high human population density. Handling of geographical data and generation of maps were performed in ArcGis version 10.2.2 ® (ESRI).

## Results

### Indicator *E*. *coli*

**Fig 2:**
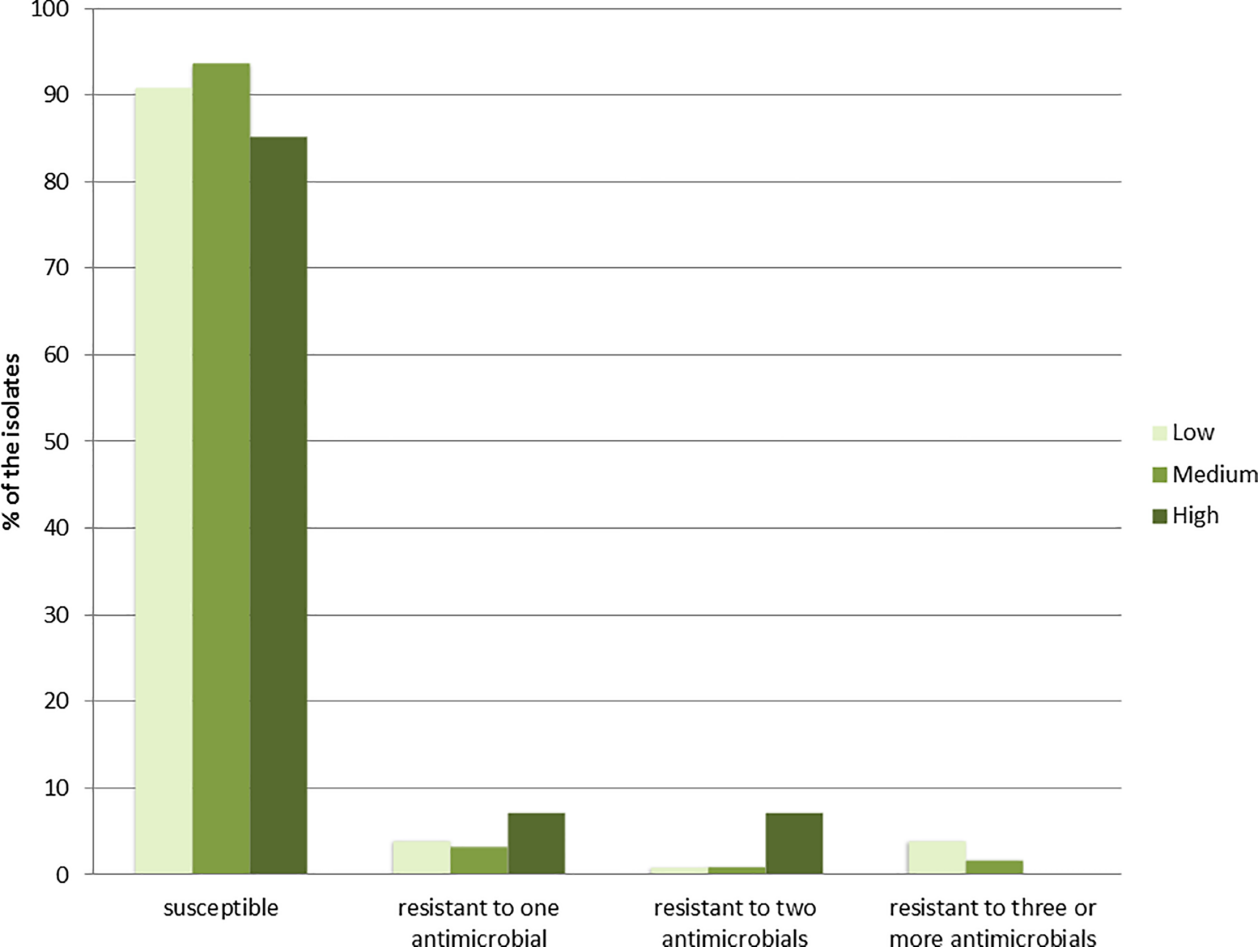
Occurrence of antimicrobial resistance among *Escherichia coli* (N = 434) isolated from wild red foxes in Norway in 2016. The isolates are categorized according to human population density in the area where the foxes were hunted, i.e. in low population density (n = 98), medium population density (n = 268) and high population density (n = 68).

*E*. *coli* were isolated from 434 of the 528 faecal samples (82.2%). Of these, 98 were isolated from samples collected in low population density areas, 268 from medium population density areas and 68 from high population density areas. The majority (91.7%) were susceptible to all antimicrobials in the panel. The occurrence of AMR (i.e. resistance to ≥1 antimicrobial) was 9.2% (95% CI: 4.3–16.7), 6.3% (95% CI: 3.7–10.0) and 14.7% (95% CI: 7.2–25.4) in the low, medium and high population density areas, respectively (Fig 2). A significant difference in AMR occurrence χ^2^ (1,N = 336) = 6.53, *p* = 0.01 was observed between medium and high population density areas. The most frequently detected resistance phenotypes among indicator *E*. *coli* were resistance to sulfamethoxazole, ampicillin and tetracycline (Table 1). Resistance to ciprofloxacin and/or nalidixic acid was only detected in isolates from medium and high population density areas (Table 1). Two indicator *E*. *coli* isolates displayed colistin resistance, but the plasmid-mediated *mcr-1* and *mcr-2* genes were not detected by PCR. Multidrug resistance (resistance to three or more antimicrobial classes) was detected in nine isolates; 2.1% (95% CI: 1.0–3.9) originating from low (n = 4) and medium (n = 5) population density areas. Resistance to cefotaxime, ceftazidime or meropenem was not detected in any of the indicator *E*. *coli* isolates.

**Table 1:** Minimum inhibitory concentrations (MICs) and antimicrobial resistance in indicator *Escherichia coli* (n = 434) isolated from faecal swab samples from wild red foxes in Norway in 2016.

		Resistance (%) [95% CI]		Distribution (%) of MIC values (mg/L)															
Substance	Area			0.015	0.03	0.06	0.125	0.25	0.5	1	2	4	8	16	32	64	128	256	≥ 512
TET	L	5.1	[1.7–11.5]								94.9					3.1	2.0		
	M	1.9	[0.6–4.3]								97.0	1.1				1.1	0.7		
	H	2.9	[0.4–10.2]								94.1	2.9				1.5	1.5		
TGC	L	0.0	[0.0–3.7]					96.9	3.1										
	M	0.4	[0.0–2.1]					97.4	2.2				0.4						
	H	0.0	[0.0–5.3]					98.5	1.5										
CHL	L	1.0	[0.0–5.6]										99.0					1.0	
	M	0.4	[0.0–2.1]										98.9	0.7				0.4	
	H	0.0	[0.0–5.3]										98.5	1.5					
AMP	L	5.1	[1.7–11.5]							1.0	30.6	53.1	10.2				5.1		
	M	2.6	[1.1–5.3]							0.7	33.6	59.0	4,1		0.4		2.2		
	H	5.9	[1.6–14.4]							1.5	33.8	51.5	7.4		1.5		4.4		
CTX	L	0.0	[0.0–3.7]					100											
	M	0.0	[0.0–1.4]					100											
	H	0.0	[0.0–5.3]					100											
CAZ	L	0.0	[0.0–3.7]						100										
	M	0.0	[0.0–1.4]						100										
	H	0.0	[0.0–5.3]						100										
MER	L	0.0	[0.0–3.7]		100														
	M	0.0	[0.0–1.4]		100														
	H	0.0	[0.0–5.3]		100														
SXT	L	5.1	[1.7–11.5]										92.9			1.0			5.1
	M	2.2	[0.8–4.8]										96.3	1.5					2.2
	H	7.4	[2.4–16.3]										91.2	1.5					7.4
TMP	L	2.0	[0.2–7.2]					87.8	9.2	1.0						2.0			
	M	1.1	[0.2–3.2]					95.1	3.7							1.1			
	H	1.5	[0.0–7.9]					86.8	10.3	1.5						1.5			
AZM	L	ND	ND								55.1	36.7	7.1	1.0					
	M	ND	ND								59.7	34.3	6.0						
	H	ND	ND								50.0	35.3	14.7						
GEN	L	1.0	[0.0–5.6]						67.3	27.6	4.1	1.0							
	M	0.4	[0.0–2.1]						70.5	26.5	2.6	0.4							
	H	0.0	[0.0–5.3]						58.8	32.4	8.8								
CIP	L	0.0	[0.0–3.7]	91.8	7.1	1.0													
	M	1.5	[0.4–3.8]	92.2	6.3			1.1					0.4						
	H	1.5	[0.0–7.9]	82.4	14.7	1.5		1.5											
NAL	L	0.0	[0.0–3.7]									99.0	1.0						
	M	1.5	[0.4–3.8]									97.0	1.5		0.4		1.1		
	H	2.9	[0.4–10.2]									97.1			1.5		1.5		
CST	L	1.0	[0.0–5.6]						1.0	98.0		1.0							
	M	0.4	[0.0–2.1]						0.4	98.9	0.4		0.4						
	H	0.0	[0.0–5.3]							98.5	1.5								

The number of isolates obtained from the areas categorised according to the human population density as Low = L; Medium = M; and High = H was 98, 268 and 68, respectively. Bold vertical lines denote epidemiological cut-off values for resistance. ND = cut-off not defined by EUCAST. CI = confidence interval. White fields denote range of dilutions tested for each antimicrobial agent. MIC values higher than the highest concentration tested are given as the lowest MIC value above the range. MIC values equal to or lower than the lowest concentration tested are given as the lowest concentration tested. TET = tetracycline, TGC = tigecycline, CHL = chloramphenicol, AMP = ampicillin, CTX = cefotaxime, CAZ = ceftazidime, MER = meropenem, SXT = sulfamethoxazole, TMP = trimethoprim, AZM = azithromycin, GEN = gentamicin, CIP = ciprofloxacin, NAL = nalidixic acid, CST = colistin.

### *E*. *coli* resistant to extended-spectrum cephalosporins (ESC), fluoroquinolones, colistin and carbapenems

By selective screening, ESC-resistant *E*. *coli* were isolated from 17 of the 528 samples; 3.4% (95% CI: 1.9–5.1). The occurrence of ESC-resistant *E*. *coli* was 1.9% (95% CI: 0.2–6.5), 3.1% (95% CI: 1.5–5.5) and 5.4% (95% CI: 1.8–12.2) in low, medium and high population density areas, respectively. There was no significant difference between the occurrence of ESC-resistant *E*. *coli* in the different areas χ^2^ (2,N = 528) = 2.13, *p* > 0.05. Only seven isolates were resistant to other antimicrobial classes; sulfamethoxazole, trimethoprim, tetracyclines, quinolones, gentamicin or tigecycline, respectively (Table 2). Multidrug resistance was observed in two isolates.

**Table 2:** Minimum inhibitory concentrations (MICs) and antimicrobial resistance in isolates of *Escherichia coli* resistant to extended-spectrum cephalosporins isolated by selective screening of faceal swabs from wild red foxes (n = 17) in 2016 in Norway.

		Distribution (n) of MIC values (mg/L)															
Substance	n resistant	0.015	0.03	0.06	0.125	0.25	0.5	1	2	4	8	16	32	64	128	256	≥ 512
TET	3								14					1	2		
TGC	1					15	1	1									
CHL	0										17						
AMP	17														17		
CTX	17							2	5	2	8						
CAZ	17							2	2	4	8	1					
MER	0		17														
SXT	4										12	1					4
TMP	3					10	4							3			
AZM	ND								5	7	3		2				
GEN	1						10	5	1					1			
CIP	3	11	3			1		1				1					
NAL	2									15						2	
CST	0							17									

Bold vertical lines denote epidemiological cut-off values for resistance. ND = cut-off not defined by EUCAST. White fields denote range of dilutions tested for each antimicrobial agent. MIC values higher than the highest concentration tested are given as the lowest MIC value above the range. MIC values equal to or lower than the lowest concentration tested are given as the lowest concentration tested. TET = tetracycline, TGC = tigecycline, CHL = chloramphenicol, AMP = ampicillin, CTX = cefotaxime, CAZ = ceftazidime, MER = meropenem, SXT = sulfamethoxazole, TMP = trimethoprim, AZM = azithromycin, GEN = gentamicin, CIP = ciprofloxacin, NAL = nalidixic acid, CST = colistin.

Isolates from low population density areas displayed an AmpC phenotype and had mutations in the promoter/attenuator region of the chromosomal *ampC* gene causing up-regulation and phenotypic ESC resistance (Table 3). Resistance not caused by chromosomal mutations was also detected in ten isolates from medium and high population density areas. We detected the following ESBL/pAmpC genes *bla*_CMY_, *bla*_CTX-M-1,_ *bla*_CTX-M-14,_ *bla*_CTX-M-15_ in the resistant isolates (Table 3). These were only detected in the high and medium population density areas. The difference was not significant (Fischer’s exact test; *p* = 0.15). The two multidrug resistant isolates harboured the *bla*_CTX-M-15_ gene. Only one of the isolates with an up-regulated chromosomal *ampC* showed additional resistance to one other antimicrobial class; sulfamethoxazole. Five of the ten (50.0%) isolates harbouring plasmid-associated genes mediating ESC resistance were shown to carry these genes on conjugative plasmids in the conjugative experiments (Table 3). Presumptive transconjugants subjected to PCR were found to harbour the expected resistance gene, confirming conjugative transfer of ESC resistance plasmids.

**Table 3:** The total numbers of the detected genotypes of extended-spectrum cephalosporin-resistant *Escherichia coli* isolated from wild red foxes in 2016 in Norway (N = 528) from areas with low, medium and high population density isolated using a selective method for detection.

Resistance genotype	Population density			Total	Conjugative transfer
	Low	Medium	High		
*bla*_CMY_		2	1	3	2
*bla*_CTX-M-1_		2		2	2
*bla*_CTX-M-14_			1	1	0
*bla*_CTX-M-15_		2	2	4	1
Up-regulated chromosomal *ampC*	2	4	1	7	NA
Total	2	10	5	17	5

The number of isolates harbouring plasmid-associated resistance genes on conjugative plasmids is indicated in the last column. NA: not applicable

**Fig 3:**
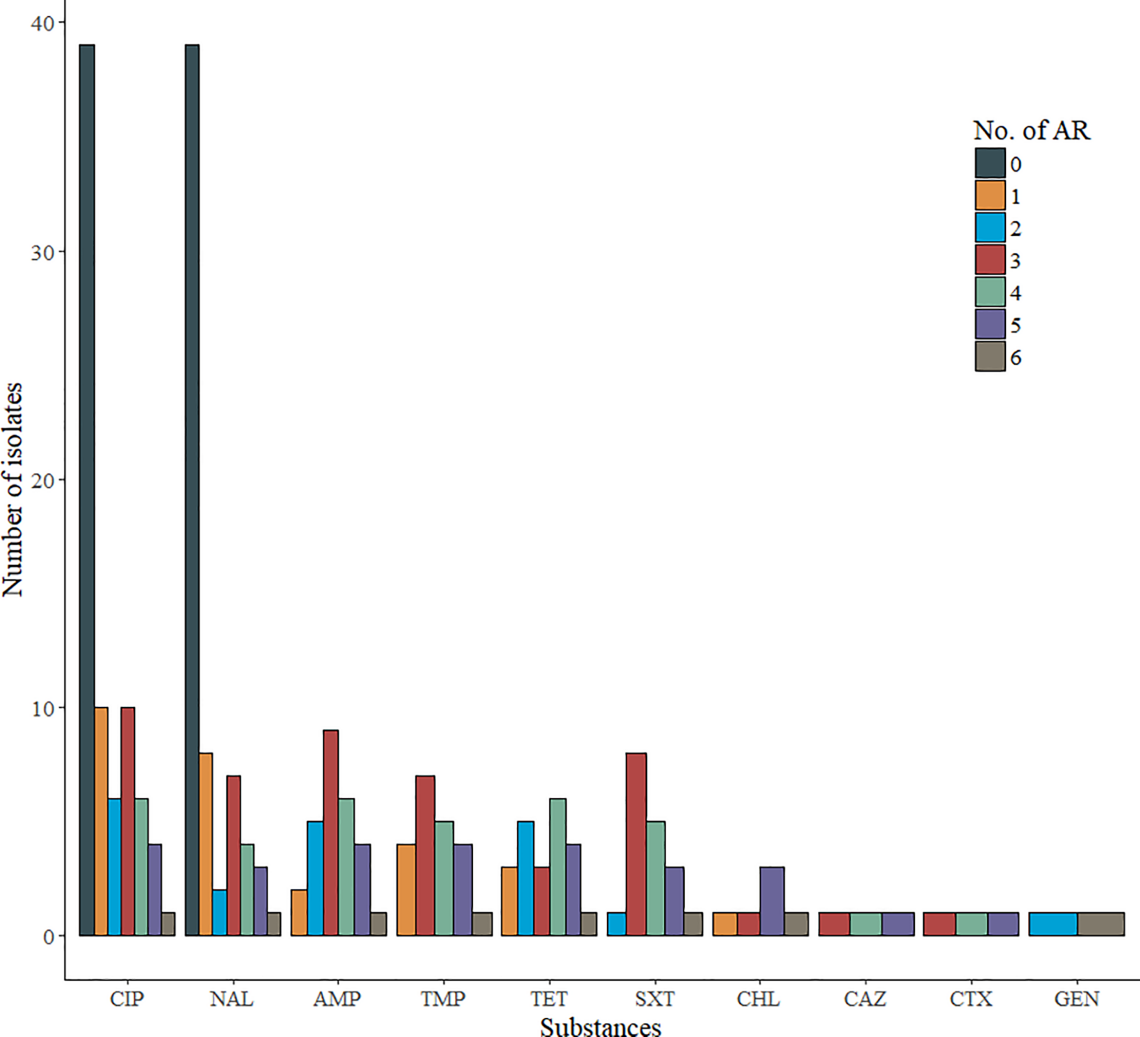
Resistance profiles of the fluoroquinolone resistant *Escherichia coli* isolates (n = 76) isolated by selective screening from wild red foxes in 2016 in Norway. No. of AR = Number of additional resistances to other antimicrobial classes than quinolones (including nalidixic and/ or ciprofloxacin), CIP = ciprofloxacin, NAL = nalidixic acid, AMP = ampicillin, TET = tetracycline, TMP = trimethoprim, SXT = sulfamethoxazole, CHL = chloramphenicol, CTX = cefotaxime, CAZ = ceftazidime, GEN = gentamicin.

Resistance to fluoroquinolones occurred in 76 of the 528 sampled foxes; 14.4% (95% CI: 11.5–17.7). The highest occurrence was found in the medium 16.1% (95% CI: 12.3–20.6) and high population density areas,15.2% (95% CI: 8.6–24.2) whereas the occurrence was 8.3% (95% CI: 3.9–15.2) in the low population density areas. There was a significant difference in the occurrence of fluoroquinolone resistant *E*. *coli* between the high and medium population density areas as compared to the low population density areas χ^2^ (1, N = 528) = 3.87, p = 0.05, whereas there was no significant difference between the high and low population density areas χ^2^ (1, N = 200) = 2.9, p = 0.09. In total, 2 of the 76 isolates; 15.7% (95% CI: 8.4–26.0) had MIC values below the ECOFF for nalidixic acid and MIC value for ciprofloxacin above the ECOFF, indicating the presence of possible plasmid mediated quinolone resistance (PMQR) genes in the isolates (Table 4). Resistance to only nalidixic acid and ciprofloxacin was observed in 39 of the 76 isolates (Fig 3). Multidrug resistance was observed in 27 of these isolates; 35.5% (95% CI: 24.9–47.3). The most common resistance profiles among these multiresistant isolates was to ampicillin followed by resistance to tetracycline, trimethoprim and sulfamethoxazole as shown in Fig 3. The phenotypic resistance profiles of these isolates showed a high diversity with 22 different resistance profiles presented in Fig 3. Further details are shown in S2 Table.

**Table 4:** Minimum inhibitory concentrations (MICs) and antimicrobial resistance in fluoroquinolone resistant *Escherichia coli* (n = 76) isolated by selective screening of faecal swab samples from wild red foxes in Norway in 2016.

			Distribution (%) of MIC values (mg/L)															
Substance	Resistance (%) [95% CI]		0.015	0.03	0.06	0.125	0.25	0.5	1	2	4	8	16	32	64	128	256	≥ 512
TET	28.9	[19.1–40.5]								71.1					6.6	22.4		
TGC	0.0	[0.0–4.7]					97.4	2.6										
CHL	7.9	[3.0–16.4]										90.8	1.3			2.6	5.3	
AMP	35.5	[24.9–47.3]							2.6	22.4	38.2	1.3			3.9	31.6		
CTX	3.9	[0.8–11.1]					96.1		1.3			2.6						
CAZ	3.9	[0.8–11.1]						96.1		1.3		1.3	1.3					
MER	0.0	[0.0–4.7]		100														
SXT	23.7	[14.7–34.8]										68.4	3.9	3.9				23.7
TMP	27.6	[18.0–39.1]					60.5	10.5	1.3						27.6			
AZM	ND	ND								40.8	34.2	18.4	2.6	2.6	1.3			
GEN	2.6	[0.3–9.2]						68.4	22.4	6.6					2.6			
CIP	100.0	[95.3–100]				14.5	57.9	7.9	10.5			5.3	3.9					
NAL	84.2	[74.0–91.6]									5.3	3.9	6.6	1.3	10.5	25.0	47.4	
CST	0.0	[0.0–4.7]							100									

Bold vertical lines denote epidemiological cut-off values for resistance. ND = cut-off not defined by EUCAST. CI = confidence interval. White fields denote range of dilutions tested for each antimicrobial agent. MIC values higher than the highest concentration tested are given as the lowest MIC value above the range. MIC values equal to or lower than the lowest concentration tested are given as the lowest concentration tested. TET = tetracycline, TGC = tigecycline, CHL = chloramphenicol, AMP = ampicillin, CTX = cefotaxime, CAZ = ceftazidime, MER = meropenem, SXT = sulfamethoxazole, TMP = trimethoprim, AZM = azithromycin, GEN = gentamicin, CIP = ciprofloxacin, NAL = nalidixic acid, CST = colistin.

*E*. *coli* displaying carbapenem or colistin resistance were not detected in the subset of investigated samples (n = 387).

### Vancomycin resistant enterococci

Enterococci displaying vancomycin resistance were not detected in the investigated samples (n = 387).

## Discussion

Overall, the occurrence of AMR in indicator *E*. *coli* from wild red foxes in Norway were low to moderate according to the definition suggested by EFSA and European Centre for Disease Prevention and Control (ECDC) [24]. The occurrence of AMR was significantly higher in areas with high population density compared to areas with medium population density. Similarly, the occurrence of fluoroquinolone resistant *E*. *coli* isolated using selective detection methods was low in areas with low population density and more common in areas with medium or high population density. Correspondingly, none of the indicator bacteria from the low population density areas displayed any resistance towards quinolones. An association between occurrence of AMR in wild animals, including small rodents and cervids, and proximity to human activity has also been suggested by others [25,26]. These findings support our hypothesis that the occurrence of AMR in wild red foxes is associated with human activity.

The association to human activity is further supported by the identification of ESC-resistant *E*. *coli* carrying plasmid-associated genes in medium and high population density areas, while ESC-resistant *E*. *coli* isolated from foxes in low population density areas only harboured chromosomal mutations leading to an up-regulation of the chromosomal *ampC* gene and phenotypic resistance. Of the ten isolates with ESBL/pAmpC encoding genes, seven harboured genes in the *bla*_CTX-M_ group. Human cases of sepsis and urinary tract infections caused by ESC-resistant *E*. *coli* are commonly associated with *bla*_CTX-M_ genes in Norway [27], while *bla*_CMY_ occurs sporadically [28]. With the exception of blaCMY that is common in broilers, these plasmid borne genes are uncommon in ESC-resistant *E*. *coli* from production animals in Norway [27]. As some of the *bla*_CTX-M_ and *bla*_CMY_ genes were located on conjugative plasmids, it may be possible that red foxes can acquire not only ESC-resistant *E*. *coli* strains, but also ESC resistance plasmids from human sources, and possibly contribute to dissemination of these AMR plasmids in the environment. However, further comparison of strains and plasmids from the two reservoirs is necessary to confirm this hypothesis.

An association with livestock production has been proposed by others, as wild animals living in areas with high livestock production have been shown to carry AMR *E*. *coli* more often than wild animals living in remote areas [29,30]. Indications of such an association are also present in our study. The most frequent resistance forms detected in indicator *E*. *coli* from wild red foxes were resistance to sulfamethoxazole, ampicillin or tetracycline. These antimicrobials are used for livestock [27]. Although the consumption of antimicrobials in the Norwegian livestock production is low [31], resistance to sulfamethoxazole, ampicillin or tetracycline are also reported in indicator *E*. *coli* from livestock, including cattle, pigs and broilers [27,32]. This indicates that livestock may be a potential source of these AMR forms in wild red foxes. However, both tetracycline and ampicillin are also commonly used in human medicine in Norway [12], and resistance towards these is also reported for human isolates. Our study categorised the areas according to the human population density and initial descriptive studies showed that the livestock production does not correlate completely with this categorisation, although there is a substantial overlap (S1 Table).

The occurrence of AMR in indicator *E*. *coli* isolated from wild red foxes was also investigated as a part of the NORM-VET programme in 2010 [33]. A limited number of samples were collected (n = 88), and *E*. *coli* were isolated from 62.5% of these (n = 55). A total of 90.9% of the isolates were fully susceptible, which is comparable to the results found in the current study. However, the panel of antimicrobials used in the current study differs from the panel used in 2010. Also, the study performed in 2010 included only a limited number of samples originating from a single county, which complicates comparison of the results. Comprehensive studies on the occurrence of AMR in wild red foxes have, to our knowledge, not been performed previously. However, other wild animals, such as lynx, wolf, wild birds, rodents and wild boars, have been suggested as reservoirs for AMR in the environment in several studies [26,34–39].

The overall occurrence of ESC-resistant *E*. *coli* was 3.2%, which is comparable with previous results reported from Portugal, where 3.8% (2/52) of foxes were found to carry ESC-resistant *E*. *coli* using a selective method for detection [40]. On the other hand, ESC-resistant *E*. *coli* were not detected in a limited number of red foxes sampled in Slovakia using a selective method [41]. However, due to minor differences in detection methods, direct comparison of the results should be made with caution.

Results from the NORM-VET programme have shown that quinolone resistant *E*. *coli* are commonly detected among several animal species when selective screening is applied [27,32]. Furthermore, a recent study on healthy volunteers in Norway revealed that almost 10% of them were faecal carriers of *E*. *coli* or *Klebsiella* spp. non-susceptible to ciprofloxacin [42]. Thus, it is not surprising that quinolone resistant *E*. *coli* are also present in red foxes, further indicating a possible spill-over of AMR from livestock production or humans to wildlife. The genetic mechanism behind the quinolone resistance in most of the quinolone resistant *E*. *coli* isolates is probably due to mutations in the quinolone resistance determining region of the chromosome located genes *gyrA*, *gyrB*, *parC* and/or *parE*. In total, 15.4% of the isolates had MIC-profiles indicating a possible plasmid-mediated resistance. However, further studies are necessary to confirm the genetic mechanisms responsible and to investigate possible routes of dissemination. Similarly, further studies are necessary to confirm the genetic mechanisms behind the colistin resistance observed among two of the indicator *E*. *coli* isolates as plasmid mediated genes, *mcr-1* and *mcr-2* were not detected. Plasmid mediated colistin resistance is uncommon from both humans and animals in Norway, indicating that the detected resistance probably is due to mutations in the chromosome.

As the samples included in this study were derived from the surveillance programme on *E*. *multilocularis*, a nationwide study was facilitated. However, as the surveillance programme is dependent on voluntary participation of hunters, a random distribution of samples cannot be expected. Thus, the number of samples included in each population density category is not equal, with most samples originating from areas with medium population density. Furthermore, an underrepresentation of certain areas was observed, such as the coastal part of southern Norway from which only a few samples were obtained. Nevertheless, the number of samples included in the study was adequate to study possible associations between human population density and occurrence of AMR in wild red foxes. An increase in the number of samples, and thereby isolates from the category “high population density”, would probably have increased the strength of the study by possibly detecting more AMR isolates within this category.

However, reasons for not detecting any multidrug resistant isolates in the category “high” population density are most probably due to the low numbers of isolates within this category, any significant difference regarding multidrug resistances was thereby not detected.

Two methods for detection of AMR bacteria were applied in the current study, namely isolation of indicator *E*. *coli* and selective detection of bacteria displaying resistance to critically important antimicrobials. Detection of AMR in indicator *E*. *coli* is an internationally established method for monitoring the occurrence and trends in AMR in Gram-negative bacteria in the intestinal flora, and gives an indication of the selection pressure exerted on the bacterial population [43]. In countries where the occurrence of AMR is low, such as Norway, it is necessary to apply selective methods in order to detect resistance towards important antimicrobials. By applying both methods, we get a better estimate of the true AMR situation in the population of wild red foxes in Norway.

In conclusion, this study shows that the occurrence of AMR in wild red foxes in Norway is associated with human population density. Foxes living in urban areas are more likely to be exposed to AMR bacteria and resistance drivers from food waste, garbage, sewage, waste water and consumption of contaminated prey compared to foxes living in remote areas. The size of red fox home ranges are varying along a gradient of productivity and human landscape alteration, generally with small sizes in agricultural areas and close to human settlements. Red fox thereby constitutes a good sentinel for monitoring AMR in the environment, with the highest geographic resolution of our results in urban areas. What impact the finding of AMR bacteriae in wild red fox has for humans is unclear. However, antimicrobial resistance may disseminate between different bacteria and species in the environment. Through a “One Health” cycle, AMR in the environment may disseminate back to humans by different means of transmission.

Continuous monitoring of the occurrence of AMR in different wild species, ecological niches and geographical areas can facilitate an increased understanding of the environmental burden of AMR. Such information is needed to further assess the impact for humans, and enables implementation of possible control measures for AMR in humans, animals and the environment in a true “One Health” approach.

## Supporting information

S1 Table Descriptive statistics of the different livestock productions in each of the human population density categories (area) in Norway in 2016. L = low, M = medium, H = high, N = Number of municipalities categorized within each of the human population density categories.
(PDF)

S2 Table Resistance profiles of the fluoroquinolone resistant *Escherichia coli* isolates (n = 76) isolated by selective screening from wild red foxes in 2016 in Norway. AR = Number of additional resistances to other antimicrobial classes than quinolones (including nalidixic and/ or ciprofloxacin). CIP_R = No. of isolates resistant to ciprofloxacin, NAL_R, AMP_R, TET_R,TMP_R,SMX_R,CHL_R, CTX_R, CFT_R, GEN_R.
(PDF)

S1 File Description of the conjugation experiments. Variables included in the file are Isolate_Nr = identification number of the isolate anonymized. Category = Human density Category, Comment; NaI = Resistant to nalidixic acid, Recipient = the isolate to which the conjugation experiment was transferred, Date = the date of experiment, 4h, 24h and 6h = result after 4 hours, 24hours and 6 hours, respectively, Bloodagar = lactose-saccharose-bromthymol blue agar plate, PCR-transconjugant detected, Date PCR = Date of performing the PCR, Final result.
(XLSX)

S2 File Data from the surveillance of antimicrobial resistance in red fox in 2016. Variables included; ID = Sample Number (anonymised, INV_NO = investigation number, SUSC_INV_NO = susceptibility test number, municipality_no = Number of the municipality, category = according to human density as described in the manuscript, Result; = 1 if agent detected; 0 otherwise, Agent_shortname = an abbreviated coded name, Agent_name = Full description of agent name,Substance = substance towards which the susceptibility testing was performed;TET = tetracycline, TGC = tigecycline, CHL = chloramphenicol, AMP = ampicillin, CTX = cefotaxime, CAZ = ceftazidime, MER = meropenem, SXT = sulfamethoxazole, TMP = trimethoprim, AZM = azithromycin, GEN = gentamicin, CIP = ciprofloxacin, NAL = nalidixic acid, CST = colistin, MIC_value = minimum inhibitory concentration values; if > a value the double value has been recorded.
(CSV)
