# Supplementary material for: What does the fox say? Monitoring antimicrobial resistance in the environment using wild red foxes as an indicator
Source: PLoS One. 2018 May 25;13(5):e0198019. doi: 10.1371/journal.pone.0198019 (PMC5969755; doi:10.1371/journal.pone.0198019)
Supplement: S1 Table — L = low, M = medium, H = high, N = Number of municipalities categorized within each of the human population density categories. (PDF) [file pone.0198019.s001.pdf]

Area	N	Livestock	Mean	StdDev	Min	Max	Median
L	147	poultry	13246.2	50777.0	0	543491	64.0
		cattle	1620.3	1750.6	0	8269	976.0
		goats	251.0	469.6	0	2443	29.0
		pig	604.7	1520.2	0	10785	14.0
		sheep	6850.0	7910.4	14	52106	4599.0
		all	15471.1	51866.1	0	552843	1893.0
M	250	poultry	57697.8	153566.3	0	947336	3878.5
		cattle	2442.7	3300.5	0	29026	1557.0
		goats	130.7	306.3	0	2867	22.0
		pig	2754.0	6518.3	0	48701	612.5
		sheep	5783.2	6961.0	3	38300	3294.5
		all	62894.4	160372.1	0	967198	8199.5
H	31	poultry	56882.7	101429.5	0	371627	273.0
		cattle	1149.9	2329.1	11	12310	533.0
		goats	23.9	56.3	0	311	6.0
		pig	1726.6	2853.7	0	11505	723.0
		sheep	2239.1	6130.9	11	34383	669.0
		all	59759.1	104837.7	23	394529	2760.0
