# Supplementary material for: What does the fox say? Monitoring antimicrobial resistance in the environment using wild red foxes as an indicator
Source: PLoS One. 2018 May 25;13(5):e0198019. doi: 10.1371/journal.pone.0198019 (PMC5969755; doi:10.1371/journal.pone.0198019)
Supplement: S2 Table — AR = Number of additional resistances to other antimicrobial classes than quinolones (including nalidixic and/ or ciprofloxacin). CIP_R = No. of isolates resistant to ciprofloxacin, NAL_R, AMP_R, TET_R,TMP_R,SMX_R,CHL_R, CTX_R, CFT_R, GEN_R. (PDF) [file pone.0198019.s002.pdf]

	No of isolates resistant to									
AR	CIP	NAL	AMP	TET	TMP	SXT	CHL	CTX	CFT	GEN
0	39	39								
1	4	4			4					
1	2	2	2							
1	2	2		2						
1	1						1			
1	1			1						
2	4		4	4						
2	1	1	1							1
2	1	1		1		1				
3	5	5	5		5	5				
3	1				1	1	1			
3	1		1	1	1					
3	1	1	1	1		1				
3	1		1	1		1				
3	1	1	1					1	1	
4	4	4	4	4	4	4				
4	1		1	1	1	1				
4	1		1	1				1	1	
5	2	2	2	2	2	2	2			
5	1		1	1	1	1	1			
5	1	1	1	1	1			1	1	
6	1	1	1	1	1	1	1			1
Total	76	64	27	22	21	18	6	3	3	2
